# Effects of Bee Pollen Derived from *Acer mono* Maxim. or *Phellodendron amurense* Rupr. on the Lipid Composition of Royal Jelly Secreted by Honeybees

**DOI:** 10.3390/foods12030625

**Published:** 2023-02-02

**Authors:** Enning Zhou, Qi Wang, Xiangxin Li, Dan Zhu, Qingsheng Niu, Qiangqiang Li, Liming Wu

**Affiliations:** 1 Apiculture Science Institute of Jilin Province, Jilin 132011, China; 2 Institute of Apicultural Research, Chinese Academy of Agricultural Sciences, Beijing 100093, China; 3 Department of Food Science, University of Otago, Dunedin 9016, New Zealand

**Keywords:** royal jelly, bee pollen, lipidomics, UPLC-Q-Exactive Orbitrap mass spectrometry

## Abstract

Royal jelly is a specific product secreted by honeybees, and has been sought after to maintain health because of its valuable bioactive substances, e.g., lipids and vitamins. The lipids in royal jelly come from the bee pollen consumed by honeybees, and different plant source of bee pollen affects the lipid composition of royal jelly. However, the effect of bee pollen consumption on the lipid composition of royal jelly remains unclear. Herein, we examined the influence of two factors on the lipid composition of royal jelly: first, two plant sources of bee pollen, i.e., *Acer mono* Maxim. (BP-Am) and *Phellodendron amurense* Rupr. (BP-Pa); secondly, different feeding times. Lipidomic analyses were conducted on the royal jelly produced by honeybees fed BP-Am or BP-Pa using ultra-high performance liquid chromatography (UPLC)-Q-Exactive Orbitrap mass spectrometry. The results showed that the phospholipid and fatty acid contents differed in royal jelly produced by honeybees fed BP-Am compared to those fed BP-Pa. There were also differences between timepoints, with many lipid compounds decreasing in abundance soon after single-pollen feeding began, slowly increasing over time, then decreasing again after 30 days of single-pollen feeding. The single bee pollen diet destroyed the nutritional balance of bee colonies and affected the development of hypopharyngeal and maxillary glands, resulting in differences in royal jelly quality. This study provides guidance for optimal selection of honeybee feed for the production of high-quality royal jelly.

## 1. Introduction

In the dietary structure of honeybees, honey and bee pollen are the main food sources. Honey is the primary source of carbohydrates, whereas bee pollen can provide a variety of nutrients for growth and development, including proteins, carbohydrates, lipids, vitamins, and minerals [1]. These substances provide bee pollen with nutritional and medical properties, such as antibacterial, antioxidant, and immunoenhancement activities [2]. Bee pollen is also an important source of proteins and lipids for production of royal jelly [3], a viscous, milky substance that is secreted by honeybee hypopharyngeal and maxillary glands. It is nutrient-rich, containing substances such as proteins, amino acids, organic acids, lipids, and minerals [4]. Royal jelly not only promotes the growth and development of queen bee larvae, prolongs the honeybee lifespan [5], benefits animal reproduction and breeding [6], but it can also serve as a human functional food and dietary supplement, and is therefore of great significance in human healthcare.

In recent years, many studies have been conducted on the nutritional components and functional activities of royal jelly. The active substances in royal jelly (such as proteins, peptides, and lipids) can endow it with various functional activities, including anti-microbial, anti-oxidative, anti-aging, anti-inflammatory, and hypoglycemic activities [7,8]. Lipids are small molecules that play important roles in maintaining the physiological dynamic balance. The LIPID MAPS database classifies lipids into eight categories, such as glycerolipids (GLs), glycerophospholipids (GPs), and sphingolipids (SPs) [9]. Royal jelly is rich in lipids, which account for 4–8% of fresh royal jelly and up to 15–30% of freeze-dried royal jelly products [3]. Free fatty acids (FAs) comprise ~90% of royal jelly lipids, which are of great significance to honeybee colonies. They not only provide nutrition for the queen and larvae during the developmental period, but also have antibacterial and mite repelling functions [10,11]. The free FAs in royal jelly also have various health-promoting activities and can be used as preventive and supplemental drugs for human health. Studies have shown that the lipid components of royal jelly can inhibit tumor cells, regulate the human immune system, prevent skin aging, and induce neurogenesis [12]. It is necessary to study the lipid composition of royal jelly to promote its development and utilization in human health applications.

The lipids in royal jelly are derived from the bee pollen consumed by honeybees. The lipid content of bee pollen is up to 13% of the dry weight, and the types and levels of lipids in bee pollen vary based on the plant source [13]. Importantly, it has been shown that the type of plant from which bee pollen is produced affects the lipid composition of royal jelly [5]. However, there has been a limited number of studies related to the effects of bee pollen consumption on the lipid composition of royal jelly. The traditional Chinese herbs *Acer mono* Maxim. and *Phellodendron amurense* Rupr. have a wide range of biological activities, including anti-oxidant, anti-tumor, anti-microbial, anti-inflammatory, anti-diabetic, and hepatoprotective effects [14,15]. Bee pollen derived from *A. mono* Maxim. (BP-Am) and from *P. amurense* Rupr. (BP-Pa) also retain these nutritional and pharmacological properties. However, the effects of BP-Am and BP-Pa consumption on royal jelly lipid composition have not been clarified.

We speculated that different plant source of bee pollen and different feeding time would significantly affect the lipid composition in royal jelly. Lipidomics technology has been widely used to identify the lipid composition in various foods and biological samples [16]. In this study, we applied a lipidomics approach to investigate the effects of two types of bee pollen (BP-Am and BP-Pa) on the lipid composition of royal jelly. Royal jelly secreted by honeybees with a natural mixed bee pollen diet was used as the control. We conducted analyses of royal jelly collected at several time points to determine changes in lipid composition during different stages of bee pollen consumption. An ultra-high performance liquid chromatography (UPLC)-Q-Exactive Orbitrap mass spectrometer was used to identify and quantify lipids in royal jelly samples. Our findings would provide guidance for the selection of the optimal feed for honeybees for the production of high-quality royal jelly.

## 2. Materials and Methods

### 2.1. Reagents

Acetonitrile (ACN), isopropanol (IPA), chloroform (CHCl_3_), ethanol, methanol (MeOH), and ammonium acetate were obtained from Fisher Scientific, Inc. (Pittsburgh, PA, USA). All solvents used as UPLC mobile phases or for lipid extraction were chromatographic grade. Other chemicals were of analytical grade and purchased from Sangon Biotechnology Co., Ltd. (Shanghai, China). Ultrapure water from a milli-Q water purification system (Millipore, Bedford, MA, USA) was used for lipidomics.

### 2.2. Bee Pollen Collection

BP-Am was collected in April 2021 (during the *A. mono* Maxim. flower season) from an apiary located at the *A. mono* Maxim. planting base in Jilin Province, China. BP-Am samples were confirmed through palynological analysis [17] to have about 82% *A. mono* Maxim. pollen grains. BP-Pa was collected in May 2021 (during the *P. amurense* Rupr. flowering season) from an apiary located at the *P. amurense* Rupr. planting base in Jilin Province, China. BP-Pa samples were confirmed to have about 86% *P. amurense* Rupr. pollen grains. Our samples met the requirements for classification as monofloral bee pollen (>80%) [18]. Bee pollen samples were ground and lyophilized into powder, sterilized by irradiation at 7 kGy, then stored at −80 °C prior to further study.

### 2.3. Royal Jelly Collection

One month before the experiment, six honeybee colonies were prepared and populations were adjusted to maintain a constant number of worker bees in each colony. Empty combs were added to each colony and stimulative feeding was performed to maintain consistent worker bee ages and larvae numbers. Royal jelly was collected from the six colonies before the honeybees were fed single bee pollen; these samples were used as the controls. Traps were then set at hive entrances to prevent honeybees from carrying pollen in. The six honeybee colonies were randomly divided into two groups of three colonies each. Each colony was regularly and separately fed with equal amounts of BP-Am or BP-Pa to ensure normal royal jelly production. An artificial royal jelly collection frame was placed into the hive. After honeybees became familiar with it, larvae were transferred into the frame to induce nurse bees to secrete royal jelly into it. Royal jelly was collected from the frame at 72 h after larvae were transferred. Royal jelly samples were stored at −80 °C prior to further study.

### 2.4. Lipid Extraction

Lipids were extracted from bee pollen and royal jelly as previously described [13]. Briefly, each 300-mg sample (BP-Am, BP-Pa, *A. mono* Maxim. royal jelly [RJ-Am], or *P. amurense* Rupr. royal jelly [RJ-Pa]) was mixed with 1.2 mL of 1:2 MeOH:CHCl_3_ (*v*/*v*). After vortexing for 5 min, 400 μL of ultrapure water was added, then samples were vortexed for 1 min. After centrifugation at 3000× *g* for 15 min, the lower organic phase was transferred to a clean glass tube and dried under a stream of nitrogen. Dried extracts were then redissolved in 200 μL of 1:2 MeOH:CHCl_3_ (*v*/*v*) for UPLC-Q-Exactive Orbitrap/MS analysis.

### 2.5. UPLC-MS Analyses

A UPLC system combined with a Q-Exactive Orbitrap mass spectrometer (Thermo Fisher Scientific, Waltham, MA, USA) equipped with a heated electrospray ionization (HESI) probe was used for lipidomic profiling as previously reported [13]. An XSelect CSH C18 column (100 × 2.1 mm, 2.5 μm) (Waters, Milford, MA, USA) was used for lipid separation. Mobile phase A was 3:2 ACN:H_2_O (*v*/*v*) and mobile phase B was 9:1 IPA:ACN (*v*/*v*), both of which contained 10 mM ammonium acetate. Previously published elution procedures and mass spectrometer parameters were used [16]. Data acquisition was performed in negative ionization mode with an *m*/*z* range of 200–2000. The resolutions of the full scan and fragment spectra were 70,000 and 17,500, respectively.

### 2.6. Data Analysis

LipidSearch v4.0 (Thermo Fisher Scientific, Massachusetts, MA, USA) was used for peak recognition, peak extraction, and lipid identification. Lipids were identified by matching to a database based on the retention time, characteristic parent ions (with an *m/z* tolerance of 5 ppm) and product ions (with an *m/z* tolerance of 8 ppm). Lipid filtration was performed using peak area and m-score thresholds of <1e^5^ and <10, respectively.

### 2.7. Statistics

One-way analysis of variance (ANOVA) was performed to test the significance of differences in lipid levels between samples using SPSS v21.0 (Statistical Product and Service Solutions, Illinois, IL, USA). Differences were considered significant at *p* < 0.05.

## 3. Results

### 3.1. Lipidomic Profiles in Different Types of Royal Jelly and Bee Pollen

Phospholipids and FAs are functional lipids that can regulate human immunity and metabolism. The composition and concentrations of functional lipids are important indexes by which food nutritional quality can be evaluated [19]. The negative ionization mode of mass spectrometry is suitable for phospholipid and FA measurements [13]. We here used negative ionization mode to analyze phospholipids and FAs in royal jelly and bee pollen samples. There were 20 kinds of ceramides (Cers), 14 kinds of phosphatidylcholines (PCs), 25 kinds of phosphatidylethanolamines (PEs), 3 kinds of sphingomyelins (SMs), and 51 kinds of FAs detected in royal jelly and bee pollen (Table S1). There were significant differences in the abundance of Cers, PCs, PEs, SMs, and monounsaturated FAs (MUFAs) between BP-Am and BP-Pa (Table 1). However, there were no significant differences in the abundance of saturated FAs (SFAs), and polyunsaturated FAs (PUFAs). We hypothesized that differences in bee pollen lipid composition may contribute to differences in the corresponding royal jelly lipid composition. Therefore, differences were analyzed in the lipid composition of two types of royal jelly (RJ-Am and RJ-Pa) obtained from honeybees fed BP-Am or BP-Pa, respectively.

### 3.2. Differences in Royal Jelly Phospholipid and Sphingolipid Composition

**Figure 1:**
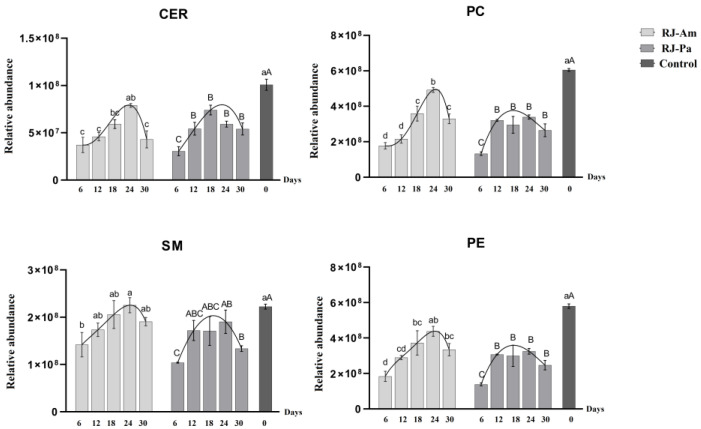
Changes in levels of phosphatidylcholines (PCs), phosphatidylethanolamines (PEs), ceramides (Cers), and sphingomyelins (SMs) in royal jelly secreted by honeybees fed bee pollen from a single plant source, *Acer mono* Maxim. (BP-Am) or *Phellodendron amurense* Rupr. (BP-Pa). Samples were collected on days 6, 12, 18, 24, and 30 after honeybees began feeding on single bee pollen. RJ-Am, royal jelly secreted by honeybees fed BP-Am; RJ-Pa, royal jelly secreted by honeybees fed BP-Pa. Significant differences in lipid content of RJ-Am or RJ-Pa secreted by honeybees fed BP-Am or BP-Pa at different timepoints are indicated with lowercase letters (a, b, c, d) and uppercase letters (A, B, C), respectively.

Phospholipids and sphingolipids are important components of cell membranes and have a variety of physiological functions that are beneficial to human health [20]. To further understand the effects of the BP-Pa and BP-Am diets on the composition of phospholipids and sphingolipids in royal jelly, we quantified SMs, Cers, PCs, and PEs in the two types of royal jelly (RJ-Am and RJ-Pa). There were clear trends in the relative content of SMs, Cers, PCs, and PEs in RJ-Am and RJ-Pa (Figure 1). In their natural state, bee colonies gather various kinds of pollen for food. After honeybees were fed with a single pollen source instead of natural pollen for six days, levels of SMs, Cers, PCs, and PEs were significantly lower in the single-pollen royal jelly compared to the control. Lipid levels in the single-pollen royal jelly began to increase after 12 days of feeding; the highest lipid levels occurred on day 18 or 24, but were not as high as lipid levels in the control royal jelly. On day 30, the lipid content of royal jelly began to decrease significantly. Notably, the average PC content was significantly higher in RJ-Am than in RJ-Pa, which might be attributed to the significantly higher PC content in BP-Am than in BP-Pa.

### 3.3. Differences in Royal Jelly Fatty Acid Composition

**Figure 2:**
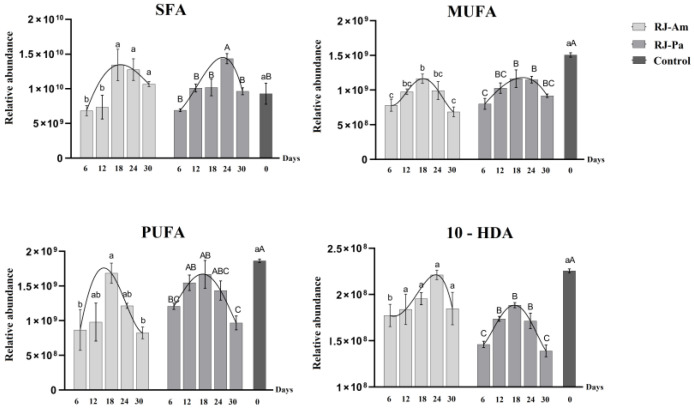
Changes in levels of saturated fatty acids (SFAs), monounsaturated fatty acids (MUFAs), polyunsaturated fatty acids (PUFAs), and 10-hydroxydec-2-enoic acid (10-HDA) in royal jelly samples. Samples were collected on days 6, 12, 18, 24, and 30 after honeybees began feeding on single bee pollen. BP-Am, single bee pollen derived from *A. mono* Maxim.; BP-Pa, single bee pollen derived from *P. amurense* Rupr.; RJ-Am, royal jelly secreted by honeybees fed BP-Am; RJ-Pa, royal jelly secreted by honeybees fed BP-Pa. Significant differences in lipid content of RJ-Am or RJ-Pa secreted by honeybees fed BP-Am or BP-Pa at different timepoints are indicated with lowercase letters (a, b, c) and uppercase letters (A, B, C), respectively.

In the present study, a relative quantitative analysis of SFAs, MUFAs, and PUFAs was carried out in royal jelly. Trends in levels of SFAs, MUFAs, and PUFAs were similar to those of SMs, Cers, PCs, and PEs (Figure 2). Specifically, SFA, MUFA, and PUFA contents were significantly decreased in royal jelly after initial feeding with single bee pollen. However, as honeybees adapted to the single-pollen diet, levels of SFAs, MUFAs, and PUFAs gradually increased. The highest levels were on day 18 or 24, but levels decreased on day 30.

In addition, trends in 10-hydroxydec-2-enoic acid (10-HDA) levels were consistent with those of other lipids in royal jelly (Figure 2): 10-HDA content was significantly decreased after initial feeding with single bee pollen; levels gradually increased as honeybees adapted to the single-pollen diet; the highest level of 10-HDA occurred on day 18 or 24; and levels were decreased on day 30. The average 10-HDA content was significantly higher in RJ-Am than in RJ-Pa. These results indicated that continuous feeding of different types of single bee pollen had significant effects on 10-HDA content in royal jelly.

**Figure 3:**
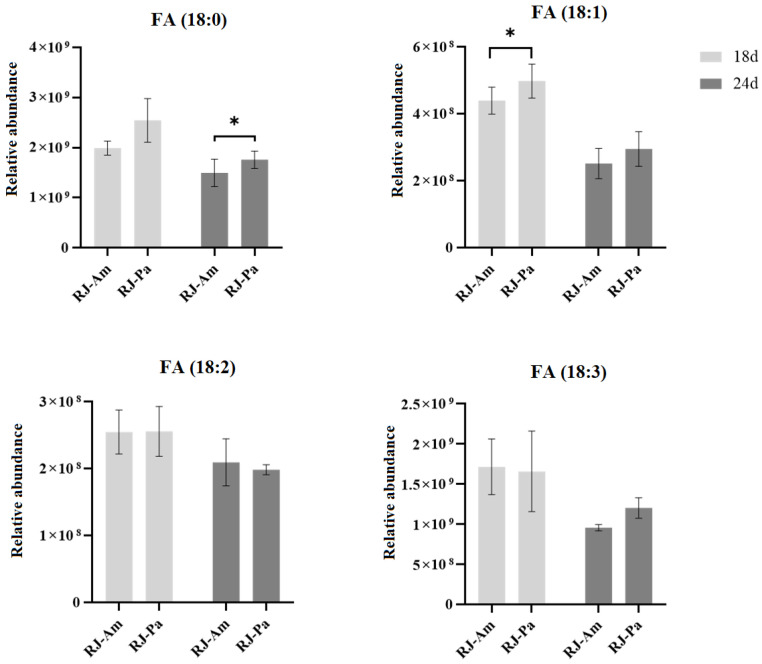
Changes in levels of 18-carbon saturated and unsaturated fatty acids in royal jelly secreted by honeybees fed single bee pollen derived from *Acer mono* Maxim. (BP-Am) or *Phellodendron amurense* Rupr. (BP-Pa). Samples were collected on days 18 and 24 after honeybees began feeding on single bee pollen. RJ-Am, royal jelly secreted by honeybees fed BP-Am; RJ-Pa, royal jelly secreted by honeybees fed BP-Pa. *, *p*-value < 0.05.

**Figure 4:**
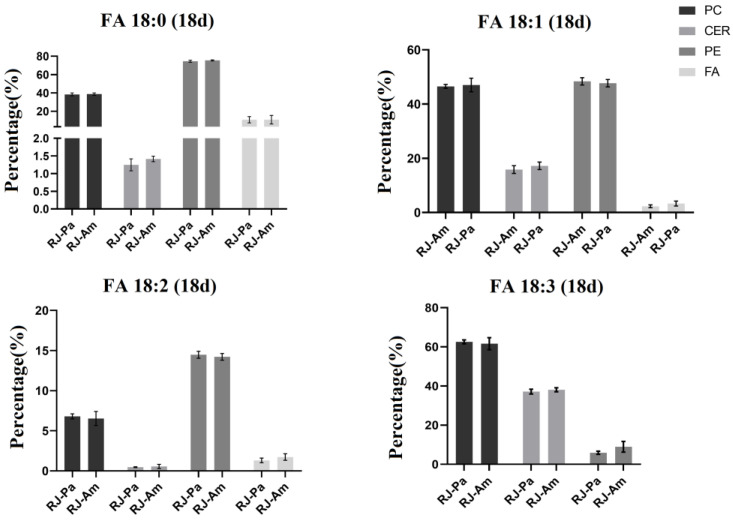
The ratio of free and bound 18-carbon saturated and unsaturated fatty acids in royal jelly secreted by honeybees fed single bee pollen derived from *Acer mono* Maxim. (BP-Am) or *Phellodendron amurense* Rupr. (BP-Pa). RJ-Am, royal jelly secreted by honeybees fed BP-Am; RJ-Pa, royal jelly secreted by honeybees fed BP-Pa.

Furthermore, we compared the relative abundance of 18-carbon fatty acids in bee pollen and found that the content of FA(18:0) and FA(18:1) in BP-Am was significantly higher than that in BP-Pa (Table 1). However, there’s no significant difference in the relative abundance of 18-carbon fatty acids between RJ-Am and RJ-Pa (Figure 3), which might be attributed to the participation of 18-carbon fatty acids in lipid metabolism and the synthesis of 10-HDA. We also analyzed the ratio of free and bound 18-carbon saturated and unsaturated FAs in RJ-Am and RJ-Pa on day 18. We found that there were no significant differences in the ratios of FA(18:0), FA(18:1), FA(18:2), or FA(18:3) between the two royal jelly samples (Figure 4). This suggested that different types of bee pollen did not affect levels of 18-carbon saturated and unsaturated FAs in royal jelly, which might be related to lipid metabolism and regulation of maxillary glands during royal jelly secretion.

## 4. Discussion

**Figure 5:**
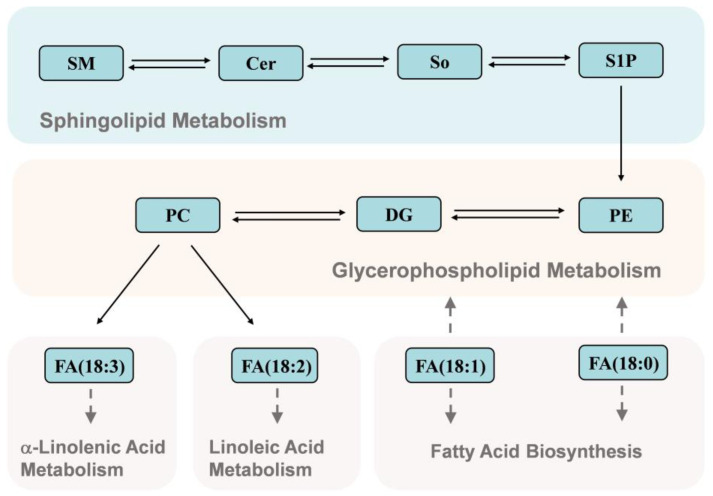
Kyoto Encyclopedia of Genes and Genomes (KEGG) biological pathway annotations for the detected lipids. SM, sphingomyelin; Cer, ceramide; So, sphingosine; S1P, sphingosine-1-phosphate; PE, phosphatidylethanolamine; DG, 1,2-diacyl-sn-glycerol; PC, phosphatidylcholine.

In this study, UPLC-Q-Exactive Orbitrap/MS analysis was conducted to explore the effects of different types of bee pollen consumption by honeybees on the lipid composition of royal jelly. PC and PE belong to the phospholipids, Cer and SM belong to the sphingolipids. PC is known as the “third nutrient” and has a variety of biological activities. It can regulate lipid metabolism, prevent vascular diseases, counteract inflammation and oxidation, improve brain and nerve function, and prevent senile dementia [21,22]. PEs maintain the structure and function of cells, repair nerve cell membranes, and promote normal metabolism in brain neurons. The PC/PE ratio is an important factor affecting metabolic dysfunction and insulin sensitivity [23]. SMs are phospholipids containing sphingosine or dihydrosphingosine; these are primarily metabolized in the small intestine and colon and have various physiological functions. For example, SM can reduce intestinal absorption of cholesterol, down-regulate proteins related to cholesterol absorption, and reduce blood cholesterol levels [24]. SMs can also promote colon cancer cell apoptosis, inhibit colon cancer cell proliferation, improve skin barrier function, and maintain physiological functions of skin [25,26]. SMs are important initial substrates of the sphingomyelin signaling pathway and can be hydrolyzed by sphingomyelinase to produce Cer. In the reverse reaction, sphingomyelinase synthesizes SMs from Cers [27]. Cer is an important signaling molecule in many basic cellular physiological and biochemical processes, such as inflammation, immune cell transport, stress responses, apoptosis, and autophagy [27]. Notably, PCs can be hydrolyzed into linoleic acid (FA 18:2) and α-linolenic acid (FA 18:3) which can participate in linoleic acid and α-linoleic acid metabolism. Stearic acid (FA 18:0) and oleic acid (FA 18:1) are also associated with glycerophospholipid metabolism through the FA biosynthesis pathway (Figure 5). Therefore, the nutritional and functional properties of royal jelly may be affected by changes in the lipid composition. Our findings suggested that the optimum time point for collecting royal jelly from artificial feeding bee pollen is 18~24 days, because the content of functional lipids in royal jelly is the most abundant during this period.

Studies have shown that honeybee foraging preferences may be related to the protein/lipid ratio in pollen. Honeybees can selectively balance their intake of amino acids and FAs during natural foraging [28]. Moreover, royal jelly is secreted by the hypopharyngeal and maxillary glands, development of which is positively correlated with the type and quantity of pollen ingested. A previous study found that consumption of mixed bee pollen is better for hypopharyngeal gland development than single pollen [29]. In addition, the increase in lipids before day 24 may therefore be attributed to a gradual adaptation to the single-pollen diet. However, the forced change from a natural mixed bee pollen diet to the single bee pollen diet may destroy the nutritional balance and therefore affect the development of honeybee hypopharyngeal glands over time, resulting in the observed decrease in lipids after 24 days of single bee pollen consumption.

Moreover, FAs can be used as fuel, bioactive lipid media precursors, and cell membrane components (in the form of phospholipids and glycolipids). SFAs do not contain unsaturated double bonds, which are generally considered the main trigger of high blood cholesterol, obesity, and coronary heart disease in humans [30,31]. Unsaturated FAs are classified as MUFAs or PUFAs, with classification depending on the number and positions of double bonds. Dietary unsaturated FAs have various physiological functions, such as inhibiting inflammatory mediators and cytokines, regulating lipid metabolism through transcription factors, and preventing cardiovascular diseases [32,33]. Ahad unique MUFA with antibacterial, antioxidative and anti-inflammatory activities, 10-HDA, accounts for over 50% of free FAs in royal jelly [12]. Honeybees that consume bee pollen from different plant sources secrete royal jelly with different levels of 10-HDA compared to those that consume single pollen [34]. In honeybee maxillary glands, 10-HDA is converted from an 18-carbon FA (stearic acid) through hydroxylation and β-oxidation to shorten the carbon chain [35]. Consumption of oleic acid can also affect 10-HDA levels in royal jelly [36]. Therefore, our results indicated that feeding bees with BP-Am was beneficial to increase the content of 10-HDA in royal jelly than feeding with BP-Pa, due to the significantly higher content of stearic acid and oleic acid in BP-Am than in BP-Pa.

## 5. Conclusions

In summary, the results indicated that levels of phospholipids and FAs were decreased in royal jelly secreted by honeybees fed with single bee pollen. However, as honeybees adapted to the single-pollen diet, levels of phospholipids and FAs gradually increased again. The highest levels appeared on days 18 or 24, but were decreased on day 30. Thus, it suggested that the optimum time point for collecting royal jelly from artificial feeding bee pollen is 18~24 days. Moreover, the average contents of PC and 10-HDA were significantly higher in RJ-Am than in RJ-Pa, indicating that continuous feeding of BP-Am was beneficial to increase the contents of PC and 10-HDA in royal jelly than BP-Pa. Additionally, our findings showed that a forced change from the natural mixed bee pollen diet to a single-pollen diet may negatively influence the quality of royal jelly. This study would provide a new perspective on bee pollen diets and scientific guidance for royal jelly production.

## Figures and Tables

**Table 1:** Relative abundance of different kinds of lipids in single bee pollen derived from *Acer mono* Maxim. and *Phellodendron amurense* Rupr.

Lipids	BP-Am	BP-Pa	*p* Value
Cer	2.89 × 10^9^ ± 3.42 × 10^8^	3.29 × 10^9^ ± 3.09 × 10^8^	0.038
PC	8.71 × 10^9^ ± 2.36 × 10^8^	5.97 × 10^9^ ± 4.18 × 10^8^	0.030
PE	1.11 × 10^10^ ± 1.94 × 10^9^	2.59 × 10^9^ ± 5.74 × 10^8^	0.012
SM	1.72 × 10^6^ ± 1.03 × 10^5^	5.60 × 10^6^ ± 5.52 × 10^4^	0.005
SFA	6.40 × 10^10^ ± 4.60 × 10^9^	3.93 × 10^10^ ± 1.72 × 10^9^	0.052
MUFA	2.94 × 10^10^ ± 5.08 × 10^9^	1.32 × 10^10^ ± 1.11 × 10^9^	0.027
PUFA	8.07 × 10^10^ ± 8.85 × 10^9^	8.64 × 10^10^ ± 3.09 × 10^9^	0.624
FA(18:0)	8.91 × 10^9^ ± 1.24 × 10^9^	2.17 × 10^9^ ± 1.66 × 10^8^	0.012
FA(18:1)	2.55 × 10^10^ ± 3.94 × 10^9^	9.87 × 10^9^ ± 8.81 × 10^8^	0.024
FA(18:2)	3.22 × 10^10^ ± 4.83 × 10^9^	2.15 × 10^10^ ± 1.04 × 10^9^	0.082
FA(18:3)	5.79 × 10^10^ ± 7.58 × 10^9^	6.66 × 10^10^ ± 4.64 × 10^9^	0.060

Note: Cer, ceramide; PC, phosphatidylcholine; PE, phosphatidylethanolamine; SM, sphingomyelin; SFA, saturated fatty acid; MUFA, monounsaturated fatty acid; PUFA, polyunsaturated fatty acid; FA(18:0), FA(18:1), FA(18:2), and FA(18:3) are classified as 18-carbon fatty acids; BP-Am, bee pollen derived from *Acer mono* Maxim.; BP-Pa, bee pollen derived from *Phellodendron amurense* Rupr.

## Data Availability

Data available on request due to privacy.

## Supplementary Materials

The following supporting information can be downloaded at: https://www.mdpi.com/article/10.3390/foods12030625/s1, Table S1: Lipidomic profiles.
