# Supplementary material for: Effects of Bee Pollen Derived from Acer mono Maxim. or Phellodendron amurense Rupr. on the Lipid Composition of Royal Jelly Secreted by Honeybees
Source: Foods. 2023 Feb 2;12(3):625. doi: 10.3390/foods12030625 (PMC9914857; doi:10.3390/foods12030625)
Supplement: Supplementary file 1 [file foods-12-00625-s001.zip › foods-2112478-supplementary.pdf]

Table S1. Lipidomic profiles

Lipid ion	m/z	Formula	Retention time (min)
Cer(d14:1_20:0)+CH3COO	596.52595	C36 H70 O5 N1	14.94
Cer(d14:1_22:0)+CH3COO	624.55725	C38 H74 O5 N1	16.41
Cer(d16:1_22:0)+CH3COO	652.58855	C40 H78 O5 N1	17.60
Cer(d18:0_16:0)+CH3COO	598.54160	C36 H72 O5 N1	15.28
Cer(d18:1_16:0)+CH3COO	596.52595	C36 H70 O5 N1	14.77
Cer(d18:1_20:0)+CH3COO	652.58855	C40 H78 O5 N1	17.21
Cer(d18:2_16:0)+CH3COO	594.51030	C36 H68 O5 N1	14.00
Cer(t18:0_16:0)+CH3COO	614.53651	C36 H72 O6 N1	14.04
Cer(t18:0_18:0)+CH3COO	642.56781	C38 H76 O6 N1	15.43
Cer(t18:0_22:0)+CH3COO	698.63041	C42 H84 O6 N1	18.35
Cer(t18:0_24:0)+CH3COO	726.66171	C44 H88 O6 N1	19.86
Cer(t18:1_16:0)+CH3COO	612.52086	C36 H70 O6 N1	13.29
Cer(t18:1_18:0)+CH3COO	640.55216	C38 H74 O6 N1	14.75
Cer(t18:1_20:0)+CH3COO	668.58346	C40 H78 O6 N1	16.10
Cer(t18:1_21:0)+CH3COO	682.59911	C41 H80 O6 N1	16.90
Cer(t18:1_22:0)+CH3COO	696.61476	C42 H82 O6 N1	17.61
Cer(t18:1_23:0)+CH3COO	710.63041	C43 H84 O6 N1	18.39
Cer(t18:1_24:0)+CH3COO	724.64606	C44 H86 O6 N1	19.10
Cer(t18:1_25:0)+CH3COO	738.66171	C45 H88 O6 N1	19.86
Cer(t18:1_26:0)+CH3COO	752.67736	C46 H90 O6 N1	20.62
PC(14:0_18:3)+CH3COO	786.52906	C42 H77 O10 N1 P1	10.78
PC(16:0_16:0)+CH3COO	792.57601	C42 H83 O10 N1 P1	14.62
PC(16:0_18:1)+CH3COO	818.59166	C44 H85 O10 N1 P1	14.67
PC(16:0_18:2)+CH3COO	816.57601	C44 H83 O10 N1 P1	13.49
PC(16:0_18:3)+CH3COO	814.56036	C44 H81 O10 N1 P1	12.31
PC(16:1_18:3)+CH3COO	812.54471	C44 H79 O10 N1 P1	11.03
PC(17:0_18:3)+CH3COO	828.57601	C45 H83 O10 N1 P1	13.08

PC(18:0_18:1)+CH3COO	846.62296	C46 H89 O10 N1 P1	16.12
PC(18:0_18:3)+CH3COO	842.59166	C46 H85 O10 N1 P1	13.96
PC(18:1_18:1)+CH3COO	844.60731	C46 H87 O10 N1 P1	14.97
PC(18:1_18:2)+CH3COO	842.59166	C46 H85 O10 N1 P1	13.43
PC(18:1_18:3)+CH3COO	840.57601	C46 H83 O10 N1 P1	12.45
PC(18:3_18:2)+CH3COO	838.56036	C46 H81 O10 N1 P1	11.27
PC(18:3_18:3)+CH3COO	836.54471	C46 H79 O10 N1 P1	10.24
PE(14:0_18:3)-H	684.46098	C37 H67 O8 N1 P1	11.08
PE(15:0_18:3)-H	698.47663	C38 H69 O8 N1 P1	11.77
PE(16:0_16:0)-H	690.50793	C37 H73 O8 N1 P1	14.61
PE(16:0_18:1)-H	716.52358	C39 H75 O8 N1 P1	14.88
PE(16:0_18:2)-H	714.50793	C39 H73 O8 N1 P1	13.79
PE(16:0_18:3)-H	712.49228	C39 H71 O8 N1 P1	12.74
PE(17:0_18:3)-H	726.50793	C40 H73 O8 N1 P1	13.33
PE(18:0_18:1)-H	744.55488	C41 H79 O8 N1 P1	16.26
PE(18:0_18:2)-H	742.53923	C41 H77 O8 N1 P1	15.18
PE(18:0_18:3)-H	740.52358	C41 H75 O8 N1 P1	14.19
PE(18:1_18:1)-H	742.53923	C41 H77 O8 N1 P1	14.83
PE(18:2_18:2)-H	738.50793	C41 H73 O8 N1 P1	12.77
PE(18:3_18:2)-H	736.49228	C41 H71 O8 N1 P1	11.58
PE(18:3_18:3)-H	734.47663	C41 H69 O8 N1 P1	10.51
PE(18:3_20:3)-H	762.50793	C43 H73 O8 N1 P1	11.83
PE(18:3_22:1)-H	794.57053	C45 H81 O8 N1 P1	15.21
PE(18:3_23:0)-H	810.60183	C46 H85 O8 N1 P1	17.33
PE(18:3_23:1)-H	808.58618	C46 H83 O8 N1 P1	15.91
PE(20:1_18:3)-H	766.53923	C43 H77 O8 N1 P1	14.00
PE(22:0_18:2)-H	798.60183	C45 H85 O8 N1 P1	17.66
PE(22:0_18:3)-H	796.58618	C45 H83 O8 N1 P1	16.66
PE(24:0_18:2)-H	826.63313	C47 H89 O8 N1 P1	19.10

PE(24:0_18:3)-H	824.61748	C47 H87 O8 N1 P1	18.06
PE(24:1_18:2)-H	824.61748	C47 H87 O8 N1 P1	17.47
PE(24:1_18:3)-H	822.60183	C47 H85 O8 N1 P1	16.57
SM(34:1)+CH3COO	761.58143	C41 H82 O8 N2 P1	13.13
SM(36:1)+CH3COO	789.61273	C43 H86 O8 N2 P1	14.72
SM(38:1)+CH3COO	817.64403	C45 H90 O8 N2 P1	16.20
FA(10:0)-H	171.13850	C10H19O2	1.17
FA(10:1)-H	169.12285	C10H17O2	1.06
FA(11:1)-H	183.13850	C11H19O2	0.82
FA(12:0)-H	199.16981	C12H23O2	1.52
FA(13:1)-H	211.16981	C13H23O2	0.92
FA(14:0)-H	227.20111	C14H27O2	2.31
FA(15:0)-H	241.21676	C15H29O2	2.97
FA(15:4)-H	233.15415	C15H21O2	1.47
FA(16:0)-H	255.23241	C16H31O2	3.92
FA(16:1)-H	253.21676	C16H29O2	2.52
FA(16:2)-H	251.20111	C16H27O2	1.86
FA(16:3)-H	249.18546	C16H25O2	1.47
FA(17:0)-H	269.24806	C17H33O2	5.14
FA(17:1)-H	267.23241	C17H31O2	3.25
FA(17:2)-H	265.21676	C17H29O2	2.30
FA(17:3)-H	263.20111	C17H27O2	1.73
FA(18:0)-H	283.26371	C18H35O2	6.58
FA(18:1)-H	281.24806	C18H33O2	4.27
FA(18:2)-H	279.23241	C18H31O2	2.89
FA(18:3)-H	277.21676	C18H29O2	2.15
FA(19:0)-H	297.27936	C19H37O2	7.92
FA(20:0)-H	311.29501	C20H39O2	9.04
FA(20:1)-H	309.27936	C20H37O2	6.88

FA(20:2)-H	307.26371	C20H35O2	4.75
FA(20:3)-H	305.24806	C20H33O2	3.33
FA(20:4)-H	303.23241	C20H31O2	2.40
FA(20:5)-H	301.21676	C20H29O2	1.97
FA(20:6)-H	299.20111	C20H27O2	2.04
FA(21:0)-H	325.31066	C21H41O2	9.95
FA(21:1)-H	323.29501	C21H39O2	8.08
FA(22:0)-H	339.32631	C22H43O2	10.89
FA(22:1)-H	337.31066	C22H41O2	9.12
FA(22:2)-H	335.29501	C22H39O2	7.36
FA(22:3)-H	333.27936	C22H37O2	5.54
FA(23:0)-H	353.34196	C23H45O2	11.82
FA(23:1)-H	351.32631	C23H43O2	9.99
FA(23:2)-H	349.31066	C23H41O2	8.51
FA(24:0)-H	367.35761	C24H47O2	12.76
FA(24:1)-H	365.34196	C24H45O2	10.86
FA(24:2)-H	363.32631	C24H43O2	9.42
FA(24:3)-H	361.31066	C24H41O2	8.07
FA(24:4)-H	359.29501	C24H39O2	6.27
FA(25:0)-H	381.37326	C25H49O2	13.68
FA(26:0)-H	395.38891	C26H51O2	14.53
FA(26:3)-H	389.34196	C26H45O2	9.93
FA(27:0)-H	409.40456	C27H53O2	15.32
FA(28:0)-H	423.42021	C28H55O2	16.14
FA(29:0)-H	437.43586	C29H57O2	16.69
FA(30:0)-H	451.45151	C30H59O2	17.81
FA(30:1)-H	449.43586	C30H57O2	15.98
FA(30:3)-H	445.40456	C30H53O2	13.45
